# Impact of advanced therapies on surgical recurrence following second ileocolic resection in Crohn’s disease

**DOI:** 10.1093/ecco-jcc/jjaf156

**Published:** 2025-08-21

**Authors:** Anouck E G Haanappel, Tycho B Moojen, Malaika S Vlug, Roel Hompes, Christianne J Buskens, Willem A Bemelman

**Affiliations:** Department of Surgery, Amsterdam UMC, Location VUmc, Amsterdam, The Netherlands; Department of Surgery, Amsterdam UMC, Location VUmc, Amsterdam, The Netherlands; Department of Surgery, Amsterdam UMC, Location VUmc, Amsterdam, The Netherlands; Department of Surgery, Amsterdam UMC, Location VUmc, Amsterdam, The Netherlands; Department of Surgery, Amsterdam UMC, Location VUmc, Amsterdam, The Netherlands; Department of Surgery, Amsterdam UMC, Location VUmc, Amsterdam, The Netherlands

**Keywords:** Crohn’s disease, ileocolic resection, disease recurrence

## Abstract

**Aim:**

Long-term outcomes and potential benefits of evolved treatment strategies in patients with Crohn’s disease (CD) undergoing a second ileocolic resection (second surgery) are not well characterized. This study aimed to evaluate the risk of a third surgery following second surgery in CD patients.

**Method:**

This retrospective cohort study included CD patients undergoing second surgery between 2000–2021 in Amsterdam UMC. Primary outcome was a third surgery due to disease recurrence at the neoterminal ileum. Two cohorts were compared to assess changes over time: C1 (2000–2009) and C2 (2010–2021).

**Results:**

In total, 110 patients were included (69 women [62.7%]; median age, 39 years [IQR 30–50]). The rates of third surgery were 12.1% at 5-years and 24.9% at 10-years. Use of prophylactic advanced therapies increased over time (C1: 16.4% vs C2: 41.7%, *P* = .004). However, the 5-year risk of third surgery was similar in both periods (C1: 12.5% vs C2: 11.5%, *P* = .45). Similarly, there was no statistically significant difference in third surgery risk between patients treated with *vs* without prophylactic advanced therapies (HR, 0.87 [95% CI, 0.37–2.02]). Most redo surgeries were performed for stricturing disease, even when the first surgery was for a different indication.

**Conclusion:**

Following second surgery, the 5-year third surgery rate is 12.1%, which has remained stable over the past two decades. No statistically significant reduction in third surgery rates were observed in patients receiving prophylactic advanced therapies. This may reflect both the predominantly stricturing disease as indication for redo surgery, which is typically less amenable to medical treatment, and the shorter follow-up in C2.

## 1. Introduction

Despite the expanding medical armamentarium for CD, up to one-third of the patients will undergo major abdominal surgery within 5 years of diagnosis.^1^ An ileocolic resection (ICR) is the most frequently performed abdominal procedure.^2^ Unfortunately, surgical resection is not curative and post-operative disease recurrence remains a significant challenge, often necessitating repeated resection of the neoterminal ileum for many patients. Ten years after primary ICR, 10–15% of the patients require one or more resections of the neoterminal ileum due to disease recurrence.^3^^,^^4^ Postoperative management has significantly evolved with the introduction of immunomodulators, anti-TNF therapy, small molecules and strict endoscopic disease monitoring, aiming to reduce the risk of recurrence. The long-term data of the LIRIC trial demonstrated that there was no surgical recurrence in the ICR-group after a median follow-up of 5 years, suggesting early surgery combined with an intensified postoperative follow-up appears to be effective in terms of reducing surgical recurrence.^5^ Despite being technically more challenging, repeated surgery is a suitable treatment option in Crohn’s disease recurrence. Previous studies have primarily focused on the safety and feasibility of a resection of the neoterminal ileum (second surgery), following first surgery (primary ICR).^6–8^ Although second surgery was associated with a higher complication rate, the incidence of major complications, such as anastomotic leakage, appeared to be low.^9^ However, data on the long-term outcomes after first redo ICR is scarce. Therefore, the present study aimed to assess the risk of surgical recurrence in CD patients who had already undergone a second surgery.

## 2. Materials and methods

### 2.1 Study design

A single center, retrospective cohort study was conducted at the Amsterdam UMC, a tertiary referral center in the Netherlands. Patients were identified in a prospectively maintained database and cross referenced with those identified through operation codes for ileocolic resection in the hospital electronic medical records. All consecutive patients ≥18 years with established CD who underwent a second surgery between January 2000 and December 2021 were included. Second surgery was defined as the first resection of the neoterminal ileum after first surgery (ileocecal resection) due to Crohn’s disease recurrence. Patients who had already undergone more than two resections of the neoterminal ileum at baseline (ie, by the year 2000) were excluded.

The Medical Ethical Committee at the Amsterdam UMC approved this study. All patients were provided with written information and given the option to withdraw from participation (opt-out).

### 2.2 Variables and data collection

Patient data were retrospectively retrieved from medical records. These included patient characteristics (sex, smoking status, age at diagnosis, Montreal classification [ie, age at diagnosis, disease location at second surgery, disease behavior at second surgery]), IBD related medication history, surgical characteristics (operation date, surgical indication, surgical approach, anastomotic technique and configuration, postoperative complication, histopathological data) and, postoperative disease recurrence.

Medication history was defined as the use of IBD-related therapies between the first and second surgery. Advanced medical therapy was defined as treatment with biologicals (such as anti-TNF agents [infliximab and adalimumab], vedolizumab, or Ustekinumab) and/or small molecules (such as Upadacitinib). Postoperative prophylactic medication use was defined as the initiation of IBD therapy within three months following the second surgery. Therapies initiated beyond 12 weeks postoperatively were not considered prophylactic, as they may reflect a clinical decision made in response to early signs of disease recurrence (symptomatic or biochemical).

The length of resected ileum was measured postoperative at the Pathology Department. Postoperative complications were defined as adverse events occurring within 30 days postoperative and graded according to the Clavien-Dindo score.^10^

Based on the first ECCO guideline recommendation to initiate prophylactic medication post-operatively in high-risk patients,^11^ two time periods were established: cohort 1 (C1) comprising patients undergoing their second surgery between 2000 and 2009 and cohort 2 (C2) containing patients from 2010 to 2021. Routine follow-up was conducted in accordance with current clinical guidelines, including standard postoperative endoscopic monitoring from 2010 onward.

### 2.3 Study outcomes

The primary outcome was the need for additional surgical resection after second surgery, defined as the need for a second resection of the neoterminal ileum (third surgery) due to disease recurrence at the neoterminal ileum and/or anastomotic site. Time to surgical recurrence was calculated as the difference in months between second and third surgery. If a temporary ileostomy was created during the second surgery, the date of ostomy reversal was used to calculate the time to surgical recurrence.

Secondary outcomes of interest were disease recurrence following second surgery necessitating balloon dilatation of the anastomosis or neoterminal ileum, or resection of another bowel segment. Two composite endpoints were defined: (i) **combined recurrence**, consisting of balloon dilatation or a third surgery; and (ii) **surgical recurrence**, consisting of balloon dilatation, a third surgery, or resection due to disease recurrence at a site other than the neoterminal ileum. Further outcomes of interest were postoperative 30-day morbidity following the third surgery, and length of ileal resection specimen. Additionally, we aimed to evaluate disease behavioral changes between second and third surgery and analyze the impact of the expanding medical armamentarium over time on the risk of a third surgery and the length of ileal resection.

### 2.4 Statistical analysis

Descriptive statistics were used to compare baseline characteristics. Categorical variables were reported as numbers and percentages, and continuous variables were summarized as mean and standard deviation (SD) for normally distributed data while for non-normally distributed data, median and interquartile ranges (IQR) are reported. Differences between C1 and C2 were analyzed using the chi-squared test or Mann-Whitney U test. The Wilcoxon signed-rank test was used to assess paired samples within the cohort. Kaplan–Meier analysis with log-rank test was performed for surgical recurrence, to calculate the surgical recurrence rates, and to evaluate differences between the two cohorts. Univariable and multivariable Cox-regression analyses were used to assess whether the time to third surgery was different between (i) C1 and C2, and (ii) prophylactic *vs* no prophylactic advanced medical therapies. The following variables were included for the multivariable Cox regression analyses: disease behavior (B3 *vs* B2), disease duration at second surgery, prior medical therapy (Immunomodulators and advanced therapies), anastomotic technique, and postoperative prophylaxis with advanced therapies. Results are expressed as (adjusted) hazard ratios with 95% CI. All tests were two-sided and *P*-values <.05 were considered statistically significant. SPPS statistics version 28.0.1.1 was used for statistical analyses. A Sankey diagram was used to present the changes in surgical indication from first surgery to second and third surgery.

## 3. Results

### 3.1 Study population characteristics

Between 2000 and 2021, 110 patients underwent a second surgery and were included in the analysis. The cohort comprised 69 females (62.7%) with a median age of 39 years (IQR 30.0–50.3). At time of second surgery, the majority of patients (71.8%) presented with stricturing disease. When grouped by time period, 60 patients were included in C1, and 50 in C2. Baseline characteristics are shown in Table 1.

**Table 1.** Baseline characteristics at second surgery.

n (%)	All patients (*n* = 110)	Cohort 1: 2000-2009 (*n = *60)	Cohort 2: 2010–2021 (*n = *50)	*P*-value
**Age at first second surgery, years**	39.0 (30.0–50.3)	39.5 (30.0–50.8)	38.0 (30.0–50.5)	0.66
**Sex, female,** *n* **(%)**	69 (62.7)	39 (65.0)	30 (60.0)	0.59
**Smoking at time of second surgery**	52 (47.3)	32 (53.3)	20 (40.8)	0.16
**Montreal Classification at time of second surgery**				
**Age at diagnosis**				0.63
A1 (<17 years)	18 (16.4)	8 (13.3)	10 (20.0)	
A2 (17–40 years)	75 (68.2)	42 (70.0)	33 (66.0)	
A3 (>40 years)	17 (15.5)	10 (16.7)	7 (14.0)	
**Location of disease at second surgery**				0.64
L1 (terminal ileum)	70 (63.6)	37 (61.7)	33 (66.0)	
L3 (ileocolonic)	40 (36.4)	23 (38.3)	17 (34.0)	
**Disease behavior at second surgery**				0.69
B1 (non-stricturing)	9 (8.2)	4 (6.7)	5 (10.0)	
B2 (stricturing)	79 (71.8)	45 (75.0)	34 (68.0)	
B3 (penetrating)	22 (20)	11 (18.3)	11 (22.0)	
**Perianal involvement**	24 (21.8)	15 (25.0)	9 (18.0)	0.38
**Medical therapy received between first and second surgery**				
Steroids	72 (65.5)	43 (71.7)	29 (58.0)	0.17
Immunomodulators	68 (63.0)	38 (63.3)	30 (61.2)	0.73
Advanced therapy	60 (54.5)	22 (36.7)	38 (76.0)	**<0.001**
**Time first to second surgery (months)**	104.5 (66.0–167.8)	90.0 (51.5–125.5)	141.0 (86.8–205.3)	**<0.001**

Data are presented as median + interquartile range (IQR), unless stated otherwise.

The median time interval from first to second surgery was 104.5 months (IQR 66.0–167.8), which significantly increased over time (C1: 90.0 months [IQR 51.5–125.5] vs C2: 141.0 [86.8–205.3]; *P* < .001).

Between the first and second surgery, most patients received treatment with steroids (65.5%), immunomodulators (63.0%) and advanced medical therapies (54.5%). Over time, the proportion of patients treated with advanced therapies (defined as treatment with biologics (ie, anti-TNF agents, vedolizumab, or Ustekinumab) and/or small molecules (eg, Upadacitinib) between first and second surgery increased significantly (C1: 36.7% vs C2: 76.0%; *P* < .001).

In addition, the number of advanced medical therapies administered between first and second surgery was significantly lower in C1 compared to C2 (median, 0.0 [IQR 0.0 to 1.0] vs 1.0 [IQR 0.5 to 1.5], *P* < .001).

Surgical characteristics of second surgery are reported in Table 2. Most procedures were performed electively (87.9%), with approximately one third conducted via laparoscopy (30.1%). Over time, the surgical approach and technique changed significantly, with an increased use of laparoscopic resections (C1: 7.0% vs C2: 58.7%; *P* < .001) and stapled anastomosis (C1: 7.4% vs C2: 93.5%; *P* < .001).

**Table 2.** Surgical data of second surgery by cohort.

	Cohort 1: 2000–2009 (*n = 60*)	Cohort 2: 2010–2021 (*n = 50*)	*P*-value
**Elective surgery**	54/58 (93.1)	40/49 (81.6)	0.082
**Surgical access, laparoscopy**	4/57 (7.0)	27/46 (58.7)	**<0.001**
**Conversion**	0/4 (0)	1/27 (3.7)	>0.99
**Fecal diversion**	5/58 (8.6)	3/48 (6.3)	0.73
**Duration of operation (hh: min)**	1:58 (1:27–2:30)	2:13 (1:54–2:52)	**0.017**
**Configuration of anastomosis** ^a^			**<0.001**
**Side-to-side**	19/54 (35.2)	44/47 (93.6)	
**End-to-end**	6/54 (11.1)	1/47 (2.1)	
**End-to-side**	29/54 (53.7)	2/47 (4.3)	
**Technique of anastomosis**			**<0.001**
**Handsewn**	50/54 (92.6)	3/46 (6.5)	
**Stapled**	4/54 (7.4)	43/46 (93.5)	
**Fistulizing disease peroperative**	18/55 (32.7)	11/45 (24.4)	0.63
**Additional procedures**	11 (19.6)	8 (17.0)	
**Stricturoplasty**	2 (3.3)	2 (4.0)	
**Segment resection**	3 (5.0)	2 (4.0)	
**Other**	6 (10.0)	4 (8.0)	
**Prophylactic postoperative therapy**	35/55 (63.6)	25/48 (52.1)	0.24
**Mesalazine**	4/55 (7.3)	1/48 (2.1)	0.37
**Steroids**	13/55 (23.6)	1/48 (2.1)	**0.001**
**Immunosuppressives**	19/55 (34.5)	5/48 (10.4)	**0.004**
**Advanced therapy**	9/55 (16.4)	20/48 (41.7)	**0.004**

Data are presented as median + interquartile range (IQR) except where indicated otherwise.

a In case of stoma placement peroperative or due to postoperative complications, technique of stoma reversal is used.

Prophylactic medical therapy was initiated in 58.3% of patients following second surgery (Table 2). Over time, treatment strategies changed significantly, with a reduction in the use of prophylactic immunomodulators use (C1: 34.5% vs C2: 10.4%; *P* = .004) and a concomitant increase in the use of prophylactic advanced therapy (C1: 16.4% vs C2: 41.7%; *P* = .004). Details regarding the specific types of advanced therapies used are provided in supplementary Table S2.

### 3.2 Need for second resection of the neoterminal ileum (third surgery)

**Figure 1.**
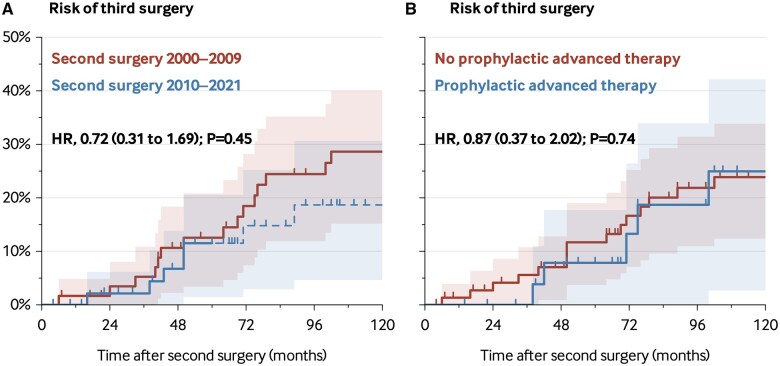
Kaplan–Meier curves for surgical recurrence following second surgery according to (A) Time period. (B) Postoperative prophylactic use of advanced therapy following second surgery.

After a median follow-up of 126 months (IQR 65.8 to 203.3), a total of 35 patients (31.8%) underwent a third surgery, with four patients (3.6%) subsequently requiring a fourth surgery during the follow-up period. The 5- and 10-year cumulative third surgery rates were 12.1% and 24.9%, respectively. The time to third surgery was similar over time, with comparable 5-year rates of third surgeries between the C1 and C2 cohorts (12.5% vs 11.5%; hazard ratio (HR), 0.72 [95% CI, 0.31 to 1.69]; *P* = .45; Figure 1A). Most patients (77/110; 70.0%) received advanced therapy at some point after the second surgery, of whom 38% (29/77) had initially received it as prophylactic treatment. The risk of a third surgery was similar between patients who received prophylactic advanced therapy initiated within three months after the second surgery, and those who did not (HR, 0.87, [0.37 to 2.02]; *P* = .74). A third surgery occurred in 21.4% of patients receiving prophylactic advanced therapy compared with 33.8% of patients who did not (*P* = .34). Prior treatment with immunomodulators or advanced therapies between the first and second surgery was significantly associated with the risk of a third surgery (HR, 2.90[1.04 to 8.08]; *P* = .04).

In multivariable analyses, prophylactic treatment with advanced medical therapies was not significantly associated with the risk of third surgery (adjusted HR, 1.10 [0.41 to 2.97]; Supplementary Table S3). However, the confidence interval was wide and compatible with both a substantially lower and substantially higher risk of third surgery with *vs* without prophylactic advanced medical therapies.

#### Disease recurrence necessitating balloon dilatation or resection elsewhere

Among the 110 patients who underwent a second surgery, 25 (22.7%) required balloon dilatation, with the majority (12/25, [48%]) undergoing a single dilatation procedure. In eleven of these 25 patients (44%), balloon dilatation was eventually followed by a third surgery. Additionally, fourteen patients (12.7%) experienced recurrence at a location other than the neoterminal ileum, necessitating further resection (eg, segmental resection/proctocolectomy), supplementary Table S1. Of these, six (42.9%) also underwent a third surgery.

The 5- and 10-year cumulative risk of combined recurrence (defined as either balloon dilatation or third resection) were 21.9% and 40.6%, respectively, for all patients. The cumulative risk of surgical recurrence (defined as balloon dilatation, third resection or resection due to disease recurrence at a site other than the neoterminal ileum), was 25.8% at 5 years and 47.2% at 10 years. No statistically significant differences were observed between cohorts for either combined recurrence and surgical recurrence (HR, 1.06, [0.56 to 2.02]; *P* = .85, and HR, 0.98, [0.54 to 1.78]; *P* = .94, respectively) (Supplementary Figures S1 and S2).

#### Characteristics patients undergoing third surgery

Of the 35 patients who underwent a third surgery, 25 (71.4%) received advanced therapy between the second and third surgery, with no significant differences between C1 (74.1%) and C2 (62.5%), *P* = .54. Laparoscopic resection was performed in fifteen patients (C1: 8/27 [29.6%] and C: 7/8 [87.5%], *P* = .011). Seven patients (20%) received an ileostomy during third surgery. Severe postoperative complications defined as Clavien-Dindo ≥ IIIa occurred in 7/35 patients (20.0%), Table 3, with no significant improvement over time, although a numerical decrease was observed (C1: 22.2% vs C2: 12.5%; *P* = 1.00). The postoperative mortality rate was 2.8%.

**Table 3.** Postoperative morbidity by cohort and surgical stage.

	Patients second surgery (*N* = 110)		Patients third surgery (*N* = 35)	
	Cohort 1: 2000- 2009 (*n = *60)	Cohort 2: 2010–2021 (*n = *50)	Cohort 1: 2000–2009 (*n = *27)	Cohort 3: 2010–2021 (*n* = 8)
**Anastomotic leakage** ^a^ **, total (** *n* **,%)**	6/55 (10.0)	3/47 (6.0)	1/20 (5.0)	0/8(0)
**Anastomotic leakage, and deviating ostomy (** *n* **,%)**	5/55 (9.1)	1/47 (2.2)	0	0 (0)
**Clavien-Dindo classification ≥ IIIa (** *n* **,%)**	10/55 (18.2)	5/49 (10.2)	6 (22.2)	1 (12.5)
**Surgical access, laparoscopy (** *n* **,%)**	4/57 (7.0)	27/46 (58.7)	8 (29.6)	7 (87.5)

a Excluding patients with diverting ostomy during surgery.

### 3.3 Disease behavioral changes

**Figure 2.**
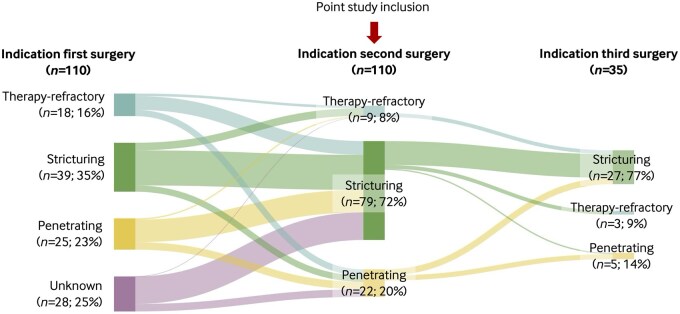
Surgical indication in our study cohort for first surgery, second surgery and third surgery.

The indication for first surgery was available for 82 patients (74.5%); among them, 22.0% underwent surgery for therapy-refractory disease, 47.6% for stricturing disease, and 30.5% for penetrating disease. In patients who initially presented with therapy-refractory disease, 16 of 18 (88.9%) progressed to complicated disease, either stricturing or penetrating, at the time of their second surgery (Figure 2). Similarly, of the patients presenting with penetrating disease at first surgery, the majority (18/25 [72%]) transitioned to a stricturing phenotype. Following consecutive resections, the proportion of patients undergoing surgery for stricturing disease increased. As a result, stricturing disease was the predominant indication for both the second (71.8%) and third (77.0%) surgery.

### 2.4 Ileal resection length

The median length of the ileal resection specimen was 25.0 cm (IQR 18.0–35.0) at first surgery, 15.0 cm (IQR 10.0–20.0) in the second surgery, and 16.0 cm (IQR 10.0–22.0) in the third surgery. The ileal resection length of the first surgery was significantly greater than at second surgery (*P* < .001). No significant reduction in ileal resection length was observed over time, Table 4. When grouped by history of advanced therapy use, the median ileal resection length at second surgery was similar in patients exposed to advanced therapy prior to second surgery and those who were not, median ileal length 15.0 cm (IQR 10.0–20.0) vs 15.5 cm (IQR 10.3–20.8), respectively, *P* = .68. Similarly, advanced therapy between second and third surgery did not result in a reduced ileal length at third (19.0 [12.0–24.3] vs 11.0 cm [IQR 8.5–18.5], *P* = .11).

**Table 4.** Median ileal resection length by cohort and surgical stage.

	Cohort 1: 2000–2009 (*n = *60)	Cohort 2: 2010–2021 (*n = *50)	*P*-value
First surgery*	20.0 cm (IQR 16.3–30.0)	28.0 cm (IQR 19.0–36.5)	0.207
Second surgery**	15.0 cm (IQR 10.0–20.0)	15.0 cm (IQR 10.0–21.0)	0.847
Third surgery***	16.0 cm (IQR 10.0–21.0)	16.0 cm (IQR 10.3–28.0)	0.627

Data available from;

* 64 patient,

** 99 patients;

*** 33 patients.

Data are presented as median + interquartile range (IQR) except where indicated otherwise.

## 3. Discussion and conclusion

This study aimed to assess long-term outcomes following the second ileocolic resection for Crohn’s disease. In this retrospective cohort of patients undergoing a second surgery, the rates of third resection at 5 and 10 years were 12.1% and 24.9%, respectively. Over the study period, treatment strategies evolved, and the proportion of patients receiving advanced therapies both prior to and as postoperative prophylaxis after the second surgery increased. However, despite these changes, the 5- year rate of third surgery was similar in both cohorts (C1: 12.5% vs C2: 11.5%, *P* = .45)

In contrast to our hypothesis, we did not observe a significant decrease in the 5-year incidence of third surgery in the most recent cohort (C2). Consistent with the guidelines and available treatment options, patients in C2 were more frequently treated with prophylactic advanced therapies following second surgery compared to C1. While the 5-year recurrence rates were similar between cohorts, it is important to note that follow-up data for C2 were sufficient only for this 5-year timeframe. Due to the truncated follow-up in C2 compared to C1, the ability to detect late recurrence is limited, which may result in an underestimation of the eventual long-term benefits of modern strategies. Therefore, the potential benefits of advanced postoperative therapies and surveillance strategies, potentially become evident with longer follow-up. In addition, no difference in the risk of third surgery was observed in patients treated with prophylactic advanced therapies. As patients who received prophylactic advanced therapies were predominantly treated in C2, it is possible that long-term effects of prophylactic advanced therapies may have been missed due to the shorter follow-up time in this group, potentially underestimating the eventual effect of advanced therapies.

It is concerning, however, that over the past two decades, no improvement has been observed in the 5-year rates of third surgery. This may be attributed to several factors. First, our findings suggest that patients who received immunomodulators or advanced therapies prior to the second surgery, which was particularly the case in the more recent cohort C2, had a higher risk of a third surgery (HR, 2.87; [1.03 to 7.98]; *P* = .043). The preoperative use of immunomodulators and advanced therapies may have selected a more severe, treatment-refractory disease group of patients. Postoperative treatment with advanced therapies could, therefore, be less effective in this cohort. Previous studies have also indicated that prior exposure to (multiple) anti-TNF-α agents before surgery is associated with reduced effectiveness of these agents in preventing postoperative disease recurrence.^12–14^ Additionally, the main surgical indication for both the second and third surgery was stenotic disease. These findings are of importance, as patients with stricturing disease are typically less responsive to medical therapies.^15^^,^^16^

As expected, our study showed a significant increase in preoperative treatment with advanced therapies and the number of different advanced therapies administered. This trend may have contributed to the prolonged interval between first and second surgery, a pattern also observed in first surgery, where anti-TNF therapy has been associated with a longer time from diagnosis to surgery.^17–19^ Although the interval from first to second surgery was longer in C2, this trend was not observed for the time between the second and third surgery. In addition, the increased use of advanced therapies did not correlate with a reduction in the length of the ileal resection specimen in either the second or third surgery. This finding is consistent with previous studies indicating that anti-TNF treatment does not reduce the extent of ileal or small bowel resection in primary surgeries.^17^^,^^18^

Repeated intestinal resections raise concerns about cumulative bowel loss, and the potential development of short bowel syndrome. Prior research has demonstrated that the presurgical extent of ileal disease correlates with the length of future recurrences.^20^ In our study, the median ileal specimen length was significantly greater at time of primary resection compared to subsequent redo procedures, consistent with previous studies reporting that the longest resection typically occurs during the initial surgery.^21^^,^^22^ Consequently, the risk of short bowel syndrome appears limited, even with repeated resections.

In this study, changes in disease behavior were observed following each subsequent resection. A substantial proportion of patients who initially underwent first surgery for perforating disease later developed a stricturing phenotype, which more frequently represented the indication for both second and third surgery. Although advanced medical therapies have been associated with reduced progression to complicated disease,^15^ these findings suggest that such treatments may prevent the development of penetrating disease but do not halt progression to stricturing disease.

Postoperative complications remain a concern for recurrent surgery. In our study, the rate of severe complications (Clavien-Dindo ≥ IIIa) following the third surgery was 20%, with higher rates observed in the earlier cohort (C1), in which open surgery was more common. Notably, the complication rate in C2, where laparoscopic techniques were more frequently used, was lower at 12.5%, consistent with previously reported short-term outcomes of third surgeries.^23^ Although redo surgery has been associated with slightly increased morbidity compared to first surgery,^23^^,^^24^ it appears that in the era of laparoscopy, redo surgery remains a feasible and safe option for patients with recurrent CD.^23–25^

To the best of our knowledge this is the first study to investigate long-term outcomes in patients undergoing second surgery for CD. One of the strengths of this study is the long follow-up duration, as it is well known that it takes time to develop a surgical recurrence. However, this study also has some limitations. First, its retrospective design which is inherent to missing data in this study. Further, potential confounding by indication may have influenced our findings, as patients perceived to be at higher risk of recurrence may have been more likely to receive prophylactic advanced therapies, potentially underestimating their true treatment effect. The impact of specific advanced therapies could not be evaluated due to small sample sizes within the respective subgroups. Additionally, the patient population represents a tertiary referral center, which may limit the generalizability of the findings to broader populations, although redo surgeries are most commonly performed in such centers. Furthermore, with only 35 patients experiencing surgical recurrence after their second surgery, the sample size may be too small to draw robust conclusions regarding the association between advanced therapy and surgical recurrence.

## Conclusion

In conclusion, this study shows that following a second surgery for CD, a third surgery is required in 12.1% and 24.9% of patients at 5 and 10 years, respectively. Over the past two decades, no statistically significant reduction in the 5-year rate of third surgeries have been observed, despite the increased use of advanced therapies. This finding may reflect the predominance of stricturing disease as indication for redo surgery, which is typically less amenable to medical treatment, as well as the shorter follow-up duration in C2. Fortunately, redo surgeries can be safely performed using minimal invasive techniques, with limited loss of small bowel. Notably, the length of ileal resection was shorter during redo surgeries compared to the first surgery.

## Supplementary Material

jjaf156_Supplementary_Data

## Data Availability

The authors of this manuscript confirm that the data supporting the results of this study are available within this manuscript. Additional details are available on reasonable request.
