# Supplementary material for: Impact of advanced therapies on surgical recurrence following second ileocolic resection in Crohn’s disease
Source: J Crohns Colitis. 2025 Aug 21;19(9):jjaf156. doi: 10.1093/ecco-jcc/jjaf156 (PMC12476910; doi:10.1093/ecco-jcc/jjaf156)
Supplement: jjaf156_Supplementary_Data [file jjaf156_supplementary_data.docx]

Supplementary tables and figures

| Table S1. Need for resection following second surgery at sites other than the neoterminal ileum | |
| --- | --- |
| N | Location resection (N=14) |
| 1x | Low anterior resection due to enterovesical fistula |
| 2x | Colostomy due to therapy refractory perianal fistula * |
| 1x | Subtotal colectomy due to descending colon stenosis |
| 2x | Proctectomy due to perianal fistula* |
| 7x | Segmental resection small bowel **** |
| 1x | Subtotal colectomy due to therapy refractory crohn’s colitis and proctectomy |
| **indicating patients who also underwent resection of the neoterminal ileum* | |

| Table S2. Specification of Prophylactic Treatment with Advanced Medical Therapies | | | |
| --- | --- | --- | --- |
| Type of prophylactic therapy | **All patients (n=29)** | **Cohort 1:**  **2000- 2009 *(n=10)*** | **Cohort 2:**  **2010-2021 *(n=19)*** |
| Anti-TNF *(Infliximab or Adalimumab)* | 19 (65.5%) | 10 (100%) | 9 (47.4%) |
| Vedolizumab | 8 (27.6) | 0 (0) | 8 (42.1%) |
| Ustekinumab | 2 (6.9) | 0 (0) | 2 (10.5%) |

| Table S3. Multivariable model of factors predictive for third surgery | | | |
| --- | --- | --- | --- |
| Variables | **n/total** | ***HR (95% CI)*** | ***P-value*** |
| Disease behaviour  B2  B3 | 69/88  19/88 | Reference  1.25 (0.45 to 3.43) | 0.67 |
| Disease duration at second surgery, per year | | 1.00 (0.94 to 1.05) | 0.88 |
| Prior medical therapy (IMMS/ advanced)  No  Yes | 20/88  68/88 | Reference  2.70 (0.72 to 10.18) | 0.14 |
| Anastomotic surgical technique  Handsewn  Stapled | 47/88  41/88 | Reference  0.53 (0.18 to 1.60) | 0.25 |
| Postoperative prophylactic advanced therapy  No  Yes | 62/88  26/88 | Reference  1.10 (0.41 to 2.97) | 0.85 |


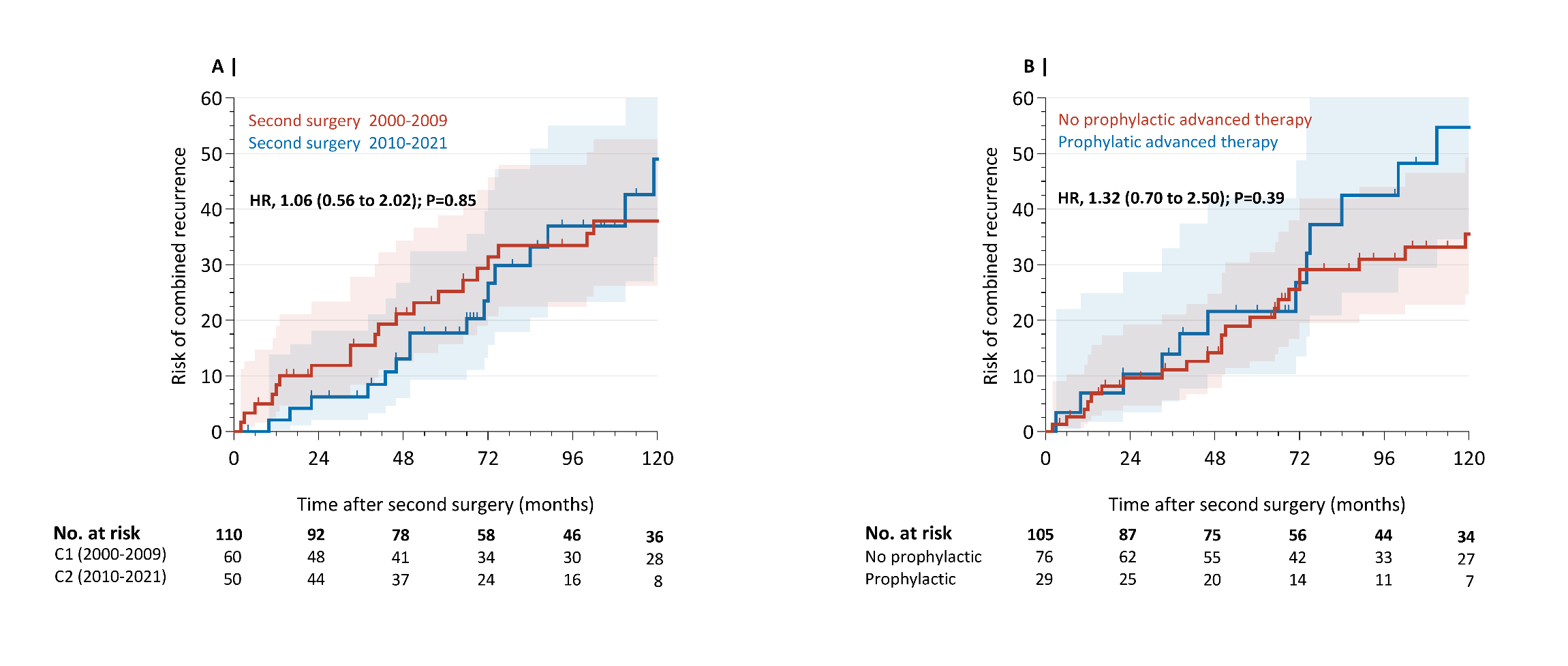


**Supplementary Figure S1**, Kaplan-Meier curves for combined recurrence (i.e. third resection and balloon dilatation) following second surgery according to

A) Time period

B) postoperative prophylactic use of advanced therapy following second surgery

**
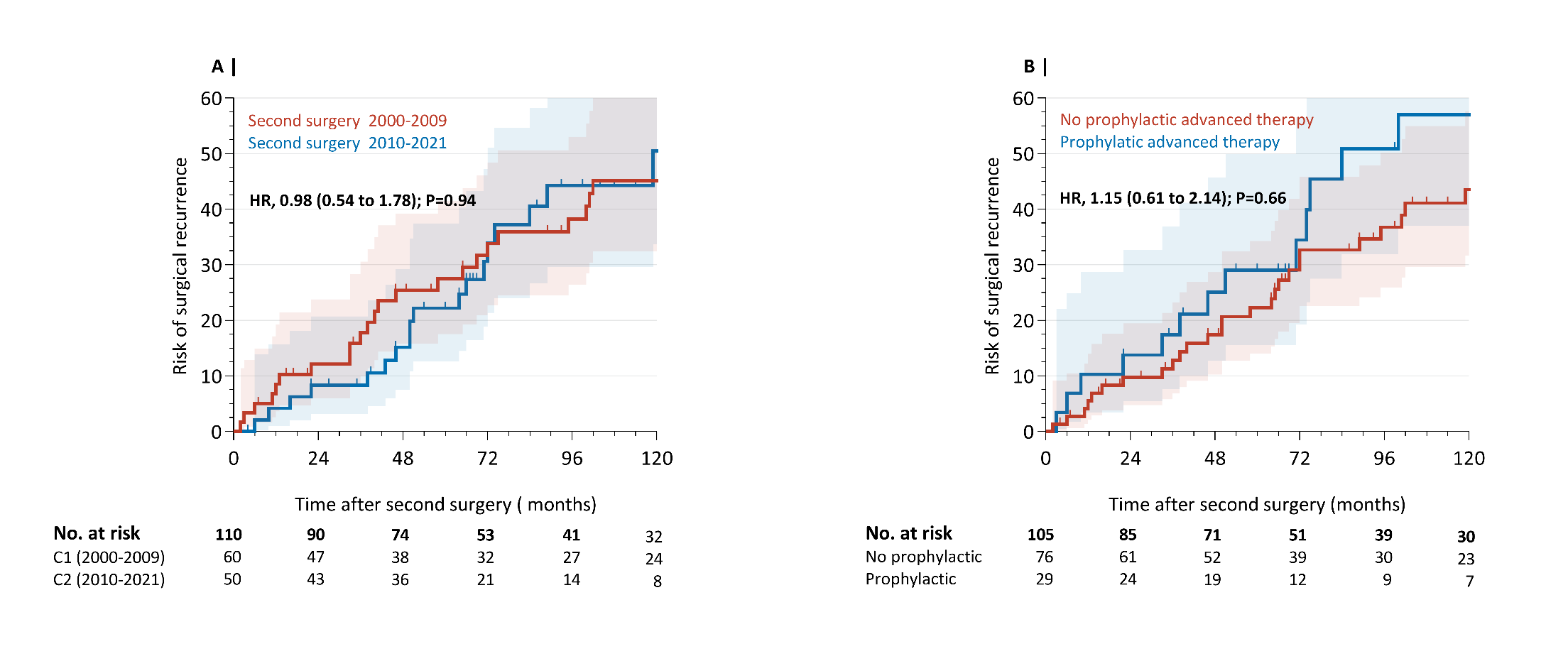
**

**Supplementary Figure S2**, Kaplan-Meier curves for surgical recurrence (i.e. third resection, balloon dilatation and resection due to recurrence at a site other than the neoterminal ileum) following second surgery according to

A) Time period

B) postoperative prophylactic use of advanced therapy following second surgery
